# CA1 Neurons Acquire Rett Syndrome Phenotype After Brief Activation of Glutamatergic Receptors: Specific Role of mGluR1/5

**DOI:** 10.3389/fncel.2018.00363

**Published:** 2018-10-17

**Authors:** Saju Balakrishnan, Sergej L. Mironov

**Affiliations:** CNMPB (Centre for Nanoscale Microscopy and Molecular Physiology of the Brain, Cluster of Excellence 171, DFG Research Center 103), Institute of Neuro and Sensory Physiology, University of Göttingen, Göttingen, Germany

**Keywords:** Rett syndrome, glutamate imaging, HCN, calcium channels, mGluR1/5, hyperexcitability

## Abstract

Rett syndrome (RTT) is a neurological disorder caused by the mutation of the X-linked MECP2 gene. The neurophysiological hallmark of the RTT phenotype is the hyperexcitability of neurons made responsible for frequent epileptic attacks in the patients. Increased excitability in RTT might stem from impaired glutamate handling in RTT and its long-term consequences that has not been examined quantitatively. We recently reported (Balakrishnan and Mironov, 2018a,b) that the RTT hippocampus consistently demonstrates repetitive glutamate transients that parallel the burst firing in the CA1 neurons. We aimed to examine how brief stimulation of specific types of ionotropic and metabotropic glutamate receptors (GluR) can modulate the neuronal phenotype. We imaged glutamate with a fluorescence sensor (iGluSnFr) expressed in CA1 neurons in hippocampal organotypic slices from wild-type (WT) and Mecp2^-/*y*^ mice (RTT). The neuronal and synaptic activities were assessed by patch-clamp and calcium imaging. In both WT and RTT slices, activation of AMPA, kainate, and NMDA receptors for 30 s first enhanced neuronal activity that induced a global release of glutamate. After transient augmentation of excitability and ambient glutamate, they subsided. After wash out of the agonists for 10 min, WT slices recovered and demonstrated repetitive glutamate transients, whose pattern resembled those observed in naïve RTT slices. Hyperpolarization-activated (HCN) decreased and voltage-sensitive calcium channel (VSCC) currents increased. The effects were long-lasting and bigger in WT. We examined the role of mGluR1/5 in more detail. The effects of the agonist (S)-3,5-dihydroxyphenylglycine (DHPG) were the same as AMPA and NMDA and occluded by mGluR1/5 antagonists. Further modifications were examined using a non-stationary noise analysis of postsynaptic currents. The mean single channel current and their number at postsynapse increased after DHPG. We identified new channels as calcium-permeable AMPARs (CP-AMPAR). We then examined back-propagating potentials (bAPs) as a measure of postsynaptic integration. After bAPs, spontaneous afterdischarges were observed that lasted for ∼2 min and were potentiated by DHPG. The effects were occluded by intracellular CP-AMPAR blocker and did not change after NMDAR blockade. We propose that brief elevations in ambient glutamate (through brief excitation with GluR agonists) specifically activate mGluR1/5. This modifies CP-AMPAR, HCN, and calcium conductances and makes neurons hyperexcitable. Induced changes can be further supported by repetitive glutamate transients established and serve to persistently maintain the aberrant neuronal RTT phenotype in the hippocampus.

## Introduction

Glutamate is the principal excitatory neurotransmitter in the brain with various functions extending far beyond glutamatergic transmission. Glutamate mediates many long-term effects, ranging from synaptic plasticity to excitotoxicity. Its functional significance is yet difficult to assess without knowing the actual levels of ambient glutamate within the nervous tissue and how fast and how much it can be changed. The main difficulties are methodological. Previous measurements of brain glutamate employed dialysis, microelectrodes, and fMRT. These methods are too slow (best temporal resolution > 1 s) and have a spatial resolution on the millimeter scale. Recent advances in the design of glutamate sensitive probes as genetically encoded fluorescent sensors (Marvin et al., 2013) provided a tool needed to quantitatively assess glutamate levels in micrometer-sized compartments at millisecond time resolution. Another valuable feature is that the sensor can be selectively embedded into neurons and astrocytes. Subsequent expression of fluorescent probe in the outer membrane leaflet can potentially report glutamate profiles in the immediate vicinity of targeted cells (Parsons et al., 2016).

We combined fluorescent glutamate measurements with calcium imaging and patch-clamp to examine glutamate-related effects in the mouse model of the Rett syndrome (RTT). This is an X-linked disorder and mainly affects girls in early childhood, causing autism-like behavior, severe intellectual disability, seizures, sleep disturbances, and autonomic instability. RTT is caused by mutations or deletion of the Mecp2 (methyl CpG-binding protein 2) transcription factor (Guy et al., 2001; Moretti and Zoghbi, 2006). Mice with Mecp2 deficiency display aberrant behavior that mirrors the symptoms in RTT patients. Mecp2 deletions have the most profound effects on synapses and impair their plasticity (Moretti and Zoghbi, 2006).

Several studies propose elevated glutamate levels in humans with RTT and mice with Mecp2 deficiency (Hamberger et al., 1992; Lappalainen and Riikonen, 1996). Johnston et al. (2014) reported chaotic sleep in Mecp2-deficient mice and correlated this with elevated baseline glutamate reinforced by prolonged wakening. How glutamate handling is related to RTT symptoms has not been examined yet. In previous studies we observed repetitive glutamate transients (spikes) in the CA1 area in RTT slices (Balakrishnan and Mironov, 2018a,b). They are based upon glutamate-induced glutamate release (GIGR) that requires intact glutamatergic transmission and increased excitability, ultimately converging onto enhanced presynaptic release of glutamate and its spillover in RTT.

Here we asked what consequences brief (30 s) stimulations of putative glutamate receptors (GluR) in the hippocampal CA1 area would have. In organotypic slices from wild-type (WT) and RTT mice, glutamate agonists to ionotropic (AMPA, kainate, and NMDA) and metabotropic receptors [(S)-3,5-dihydroxyphenylglycine (DHPG)] augmented neuronal activity transiently, which increased extracellular glutamate. Brief augmentation of activity was followed by depression. Intriguingly, after wash-out, new activity patterns were established that persisted for several hours. In WT slices, repetitive glutamate spikes appeared with the pattern that strikingly resembled the one observed in naïve RTT slices (Balakrishnan and Mironov, 2018a,b; and this study). Single application of GluR agonists in RTT slices potentiated repetitive activity that existed previously. We further applied whole-cell patch-clamp to record excitatory postsynaptic currents (EPSC), back-propagating potentials (bAPs), and voltage-dependent ion currents [hyperpolarization-activated (HCN); and the voltage-sensitive calcium channels (VSCC)]. After GluR agonists the amplitude of HCN currents decreased and VSCC currents increased. In WT the changes were bigger and the characteristics of the channels became close to those recorded in naïve RTT slices. The postsynaptic changes were assessed with a non-stationary noise analysis of EPSCs and back-propagating action potentials (bAPs). After GluR agonists the mean single channel conductance and number of channels increased. Pharmacological evidence indicates the appearance of additional calcium-permeable CP-AMPA receptors (CP-AMPAR). This mechanism should further enhance excitability that we indeed observed in recording bAPs.

The main finding of the study is that, despite distinct differences between WT and RTT networks in naïve slices, WT can seemingly acquire the RTT phenotype by a single stimulation with GluR agonists. We recently showed (Balakrishnan and Mironov, 2018b) that acidic pH and Mg^2+^ shift the voltage-dependent activation of HCN and VSCC channels in RTT that dampens excessive excitability and produces WT phenotype. The effects are reversible and do not persist at return to the normal pH. In this study we saw transformation of WT into RTT phenotype after GluR stimulation and the effects were maintained for hours. We focused on metabotropic glutamate receptors (mGluR1/5), because they are activated by submicromolar glutamate and the cytoplasmic pathways downstream exert long-lasting effects. These features make them an ideal candidate to translate brief elevations in ambient glutamate into the long-term modifications in neuronal activity. The results obtained tempt to speculate that mGluR1/5 can operate as a dispatcher that regulates a transition from WT phenotype to RTT. A suggestion is supported by recent data underscoring the role of mGluRs in RTT (Gogliotti et al., 2016; Tao et al., 2016). If mGluR1/5 activity could be dampened, RTT phenotype could be possibly reversed to a WT one. This could be used to develop a simple and reliable tool to correct RTT and perhaps hinder its development.

## Materials and Methods

### Tissue Preparation

All animals were housed, cared for and euthanized in accordance with the recommendations of the European Commission (No. L358, ISSN 0378–6978), and protocols were approved by the Committee for Animal Research, University of Göttingen. The experiments were performed using the mouse model for RTT; strain B6.129P2(C)- MeCP2tm1-1Bird (Guy et al., 2001) obtained from the Jackson Laboratory (Bar Harbor, ME, United States). Before preparation, the mice were routinely genotyped in accordance with the Jackson Laboratory genotyping protocols allowed to unequivocally distinguish between the WT and MECP2-null (RTT) mice.

Organotypic slices were prepared using the procedure developed by Stoppini et al. (1991) with modifications applied previously to the brainstem (Hartelt et al., 2008; Mironov et al., 2009, 2011) and hippocampal slices (Toloe et al., 2014; Balakrishnan and Mironov, 2018a,b). In summary, the two hippocampi from the animals at postnatal day P3 were isolated; 12–18 transverse 250-μm thick slices were cut off and placed on the support membranes (Millicell-CM Inserts, PICMORG50; Millipore). One milliliter of medium was added to have the surface of the slice continuously exposed to carbogen (5% CO_2_ in O_2_) and the medium (50% MEM with Earle’s salts, 25 mM HEPES, 6.5 mg/ml glucose, 25% horse serum, 25% Hanks solution buffered with 5 mM Tris and 4 mM NaHCO_3_, pH 7.3) was changed every 2 days. All salts and other common chemicals were from Sigma (Deisenhofen, Germany). The agonists and antagonists of channels and receptors were from Tocris Bioscience (Bristol, United Kingdom). The stock solutions of drugs were made either in DMSO or artificial cerebrospinal fluid (ACSF).

Slices for experiments were virally transduced with glutamate sensor (Marvin et al., 2013) targeted to neurons (AAV5.hSyn.iGluSnFr.WPRE.SV40) or astrocytes (AAV5.GFAP.iGluSnFr.WPRE.SV40). The constructs were purchased from Penn Vector Core (Department of Pathology and Laboratory Medicine; University of Pennsylvania, Philadelphia, PA, United States). Transduction was performed 2 days after plating slices. The experiments were performed from P7, after expression of sensors was uniform across the slice. As in our previous studies with other genetically encoded sensors (Hartelt et al., 2008; Mironov et al., 2009, 2011; Toloe et al., 2014), no morphological and electrophysiological differences of transduced neurons in comparison with their naive counterparts were noticed.

For the experiments, the membrane with the attached slice was fixed on a coverslip in the recording chamber and continuously superfused at 34°C with ACSF containing (in mM): 138 NaCl, 3 KCl, 1.5 CaCl_2_, 1 MgCl_2_, 30 HEPES, 1 NaH_2_PO_4_, 10 glucose, and pH 7.4. The volume of the perfusion chamber was 2 ml and the solution was delivered at a flow rate of 10 ml/min. Solutions were exchanged by replacement of a distal reservoir with another one that contained drugs. New solutions arrived at the slice within 30 s.

### Imaging

The cells were viewed under a 40× objective (LUMplan, N. A. 0.8). The optical recording system included an upright microscope (BX51, Olympus, Hamburg, Germany) equipped with a monochromatic light source (CAIRN, United Kingdom). Images from a cooled CCD camera (ANDOR) were digitized (256 × 256 pixels at 12 bit resolution) and collected with ANDOR software. MetaMorph (Princeton Instruments) was used for offline analysis. For measuring glutamate increases with a better time resolution a iXon Ultra 888 camera (Belfast, United Kingdom) was used. The image was cropped to 80 × 80 pixels and the acquisition time for a single frame was from 0.5 to 2 ms. For glutamate imaging, iGluSnFr was excited at 470 nm and the emission was collected at 525 ± 10 nm (49002, Chroma Technology, Olching, Germany). In a set of experiments we imaged intracellular calcium transients simultaneously with patch-clamp recordings. We used either bulk loading of slices with Fura-2 AM (10 μM, 30 min at 37°C) or filled the patched cell with 100 μM Fluo-4. To visualize neuron morphology, Alexa 568 (100 μM) was added to patch pipette. Alexa was excited at 565 nm and excitation/emission light was separated with dichroic mirror with mid-transmission at 585 nm (T585lpxr, Chroma Technology, Olching, Germany). Glutamate levels were obtained from relative increases in fluorescence Δ*F/F_0_* through inversion the Michaelis–Menten-like equation Δ*F/F_0_* = *F_max_*/(*K_d_* + [*Glu*]). Here *F_0_* is the resting (background-subtracted) fluorescence and the values *F_max_* and *K_d_* = 10 μM were obtained from calibrations with preset [Glu] in the bath. The mean responses were calculated for 12–20 CA1 neurons in the image field (the SEM in all Figures is shown by light gray background).

To monitor intracellular calcium, we used Fluo-4. The sensor was excited at 470 nm, and emission was collected at 520 nm (49002, Chroma technology, Olching, Germany). Close correspondence between patch-clamp measurements of the calcium current and Fluo-4 imaging is illustrated by **Supplementary Figures S1, S2**.

### Electrophysiology

Patch electrodes from borosilicate glass (WPI, Berlin, Germany) had resistances 2–3 MΩ when filled with K^+^-gluconate (110 mM), KCl (5), HEPES (50), EGTA (0.005), MgSO_4_ (4), ATP (4), GTP (0.2), phosphocreatine (9), and pH to 7.4 with 1 M KOH. For HCN and calcium current recordings we used a Cs^+^ based solution contained CsMeSO_4_ (92 mM), CsCl (43) TEA-Cl (5), EGTA (0.4), MgCl_2_ (1), HEPES (10), ATP (4), GTP (0.4), pH to 7.4 with CsOH. The HCN currents and VSCC currents were evoked by voltage steps to -110 and -50 mV, respectively, from a holding potential of -70 mV (close to the resting potential measured in CA1 neurons). The inclusion of Cs^+^ and TEA^+^ (Tetraethylammonium) in the intracellular solution excluded the outward K^+^ current completely. Hyperpolarization-activated (I_HCN_) and depolarization-evoked calcium (I_Ca_) currents were activated below -70 and above -60 mV, respectively, and clearly isolated. Membrane current and voltage were recorded with a patch-clamp amplifier EPC-9 (HEKA, Germany) as described previously (Balakrishnan et al., 2016). Membrane currents were filtered at 3 kHz (-3 dB), digitized at 10 kHz, and stored for off-line analysis. bAPs were evoked by trains of eight pulses at 100 Hz by injection of slightly suprathreshold currents through the patch electrode.

Analysis of spontaneous postsynaptic potentials (sEPSC) was made as described previously (Mironov and Langohr, 2007). The first two moments m_n_ = ∫ *I*^n^ (*t*) *dt* of the continuous 10 s-long-recordings of the current *I*(*t*) at -70 mV were calculated after bursts were excluded. The mean amplitude (*A*) and frequency of synaptic currents (*f*) were determined as *A* = 4τ*m*_2_/*m*_1_ and *f* = *m*^2^/4τ*m*_1_ where τ is a decay constant determined from its mean EPSC waveform.

### Non-stationary Noise Analysis

The data was analyzed with custom software written on Pascal. First, single, non-overlapping EPSCs with the fast rise time were isolated. An ensemble of single events was aligned by the point of maximal rise, averaged and scaled to the peak. After subtraction of mean EPSC (*I*) from individual responses we obtained the variance (*σ^2^*), fitting it with the equation *σ^2^ = iI -I^2^/N* + $\sigma_{0}^{2}$ (Hille, 2001). Here *i* is the single channel current, *N* is the number of channels, and $\sigma_{0}^{2}$ is the background variance.

### Statistics

Approximately equal numbers of neurons were measured in parallel both for the WT and MeCP2-null mice and analyzed blinded to genotype. Each test performed in this study was repeated for at least three different slice preparations and means ± SEM were compared by using Student’s *t*-test, with *p* < 0.05 being the criterion for statistical significance. The results of independent experiments were compared with Mann–Whitney U test. The traces in figures show typical responses overlaid upon thick gray areas that indicate variation between single traces in imaging.

## Results

**FIGURE 1:**
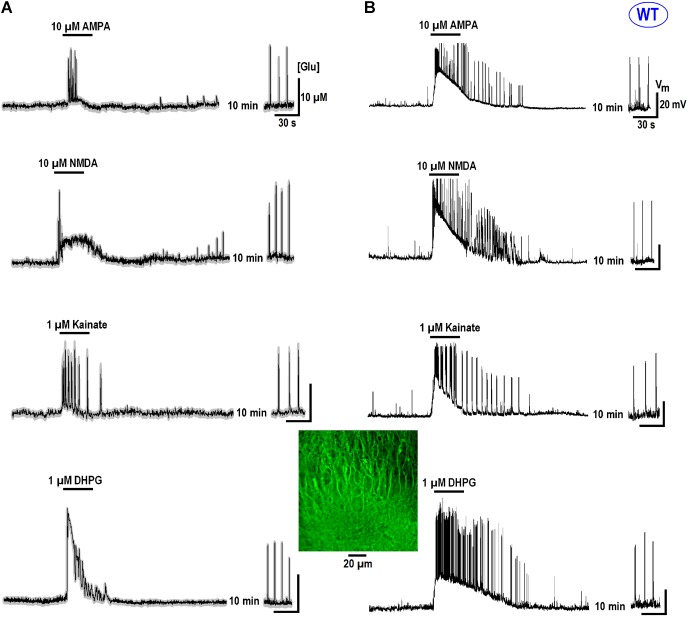
GluR agonists transiently increase ambient glutamate in CA1 neurons and induce repetitive glutamate spikes in WT. **(A)** Changes in ambient glutamate (mean traces were obtained from 12 regions of interest that encircled the neurons expressing iGluSnFr). Average traces are overlaid onto gray background showing ± SEM. **(B)** One neuron in each image field was patched and the traces on the right present representative current-clamp recordings. The ambient glutamate changes on the left and voltage trajectories on the right were measured simultaneously. In naïve slices from WT animals, repetitive glutamate spikes were observed only in 14 from 112 slices examined (see also the insets in **Figure 4**). After recovery, the regular spikes appeared in WT (continuation of traces after 10 min wash-out). The calibration bar scales are indicated only in the first panel. The inset (middle bottom) shows a typical structure of CA1 layer in WT. Group summary is given in **Table 1** that presents durations of augmentation and depression for global glutamate transients, and the amplitudes and frequency of glutamate spikes and neuronal bursts 10 min after the application of GluR agonists.

**FIGURE 2:**
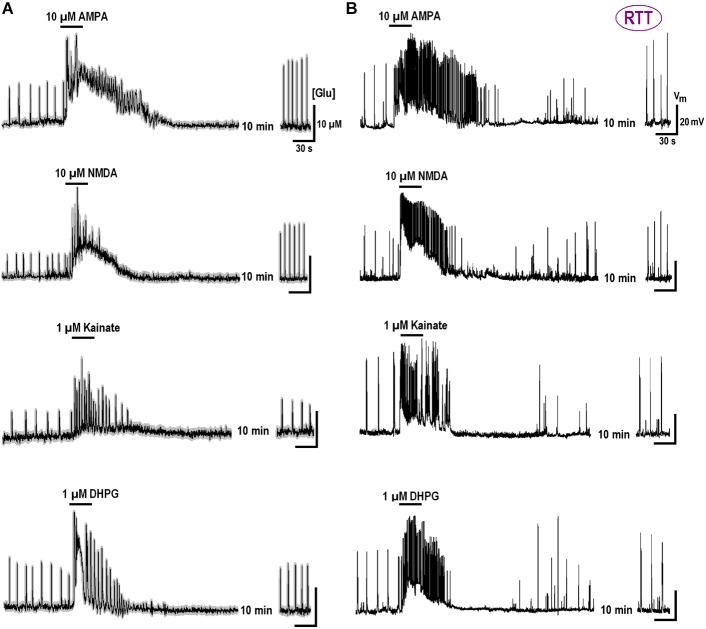
Glutamate release in CA1 cells from the MeCP2-null animals (RTT) by GluR agonists and potentiation of repetitive activity after recovery. The protocols were the same as in the experiments presented in **Figure 1**. Naive RTT slices demonstrated regular glutamate spikes. GluR agonists induced glutamate release **(A)** after increase in neuronal activity **(B)**. After brief augmentation of activity was depressed and restored after wash-out for several minutes. The glutamate spikes reappeared at significantly higher frequencies and amplitudes. The calibration bars apply for all traces and the scales are indicated only in the first panel. **Table 2** gives the durations of global glutamate release and depression phases, and amplitude and frequency of glutamate spikes and bursts before and 10 min after the application of GluR agonists.

All data in this study was collected in organotypic hippocampal slices expressing the glutamate sensor iGluSnFr (in the section “Materials and Methods”). In RTT we consistently observed spontaneous glutamate transients (spikes) coinciding with the bursts of action potentials (AP), in line with previous studies (Balakrishnan and Mironov, 2018a,b). Glutamate agonists elevated ambient glutamate to 4–10 μM for ∼10 s in WT and to 15–25 μM for ∼20 s to RTT slices (**Figures 1, 2**, left panels, summaries in **Tables 1, 2**) that paralleled the changes in neuronal activity (**Figures 1, 2**, right panels). The responses were stereotypic in both WT and RTT slices and consisted of a global increase in ambient glutamate with spontaneous glutamate spikes on the top, followed by depression. Significantly bigger and longer lasting increases in RTT slices can be explained by diminished glutamate uptake (Balakrishnan and Mironov, 2018a). Durations of depression phases in WT and RTT did not differ considerably. After washout of the agonists for 10 min (the traces in **Figures 1, 2** after the pause), the activity recovered. The pattern was markedly different from that in the control. In WT slices the repetitive glutamate spikes appeared and they looked akin to those observed in naïve RTT slices. GluR agonists in RTT reinforced spontaneous glutamate transients. The mean frequencies and amplitudes of glutamate spikes are listed in **Tables 1, 2**.

**Table 1:** Characteristics of responses elicited by glutamate agonists in CA1 neurons in slices prepared from wild-type (WT) animals.

		AMPA, 10 μM	NMDA, 10 μM	Kainate, 1 μM	DHPG, 1 μM
Global glutamate transient	Peak [Glu], μM	3.5 ± 0.4	6.7 ± 0.6	4.6 ± 0.4	9.5 ± 0.7
	Duration, half-width, s	9.5 ± 0.8	16.7 ± 0.7	7.6 ± 0.5	11.5 ± 0.6
Depression of bursts, s	Duration, s	33.5 ± 2.7	25.7 ± 2.6	44.6 ± 2.4	27.5 ± 1.7
Delay between APs generation and [Glu] increase, s		3.5 ± 0.6	4.1 ± 0.8	3.2 ± 0.4	5.3 ± 0.7
Glu spikes after agonist	Amplitude, μM	12.5 ± 1.3	11.7 ± 2.4	13.6 ± 1.2	19.5 ± 1.9
	Interval, s	14.5 ± 1.5	10.7 ± 1.4	14.6 ± 1.5	9.5 ± 0.9
Bursts after agonist	Interval, s	13.7 ± 1.3	11.0 ± 1.2	13.9 ± 1.0	10.2 ± 0.8
Number of tests		6	4	5	12


*All tests were made in different fresh (naive) preparations. The third row indicates a delay between the beginning in neuronal activity and increase in ambient glutamate (measured at half-rise-time). Mean data ± SEM were obtained from experiments whose number is indicated in the last row. For analysis we used the data obtained in naive WT slices that showed no spontaneous glutamate transients. They were established ∼10 min after wash-out of GluR agonists. For all agonists, the mean interspike intervals coincided with interburst periods (*p* > 0.1, Student’s *t*-test).*

**Table 2:** Characteristics of responses elicited by glutamate agonists in CA1 neurons in slices prepared from RTT animals.

		AMPA, 10 μM	NMDA, 10 μM	Kainate, 1 μM	DHPG, 1 μM
Global glutamate transient	Peak [Glu], μM	17.6 ± 1.4	20.0 ± 1.5	19.9 ± 1.6	26.0 ± 1.1
	Duration, half-width, s	19.1 ± 1.8	18.7 ± 1.4	17.6 ± 1.5	21.5 ± 1.6
Depression of bursts, s	Duration, s	31.5 ± 2.2	35.7 ± 2.2	34.6 ± 2.4	29.5 ± 2.7
Delay between APs generation and [Glu] increase, s		2.5 ± 0.4	3.1 ± 0.7	2.9 ± 0.5	4.3 ± 0.6
Glu spikes before agonist	Amplitude, μM	11.6 ± 1.0	11.5 ± 1.4	10.7 ± 1.6	9.1 ± 0.5
	Interval, s	15.5 ± 1.3	14.7 ± 1.5	15.6 ± 1.7	16.2 ± 1.2
Bursts before agonist	Interval, s	15.7 ± 1.4	14.0 ± 1.3	14.9 ± 1.3	15.2 ± 1.1
Glu spikes after agonist	Amplitude, μM	19.8 ± 1.2	19.4 ± 1.2	18.9 ± 0.6	19.5 ± 0.6
	Interval, s	10.5 ± 1.2	9.7 ± 1.0	10.6 ± 1.1	9.1 ± 0.8
Burst after agonist	Interval, s	10.7 ± 1.1	10.0 ± 1.0	9.9 ± 0.9	9.2 ± 0.7
Number of tests		5	4	6	8


*All tests were made in different fresh (naive) preparations. The third row presents a mean delay between increases in neuronal activity and ambient glutamate (measured at half-rise-time). Mean data ± SEM were obtained from experiments whose number is indicated in the last row. The means for global glutamate transients measured in WT and RTT (first three rows in **Tables 1, 2**) are significantly different at *P* < 0.05 (Mann–Whitney U test). The amplitudes and interspike intervals before and after application of GluR agonists are significantly different at *p* < 0.05 (Student’s *t*-test).*

**FIGURE 3:**
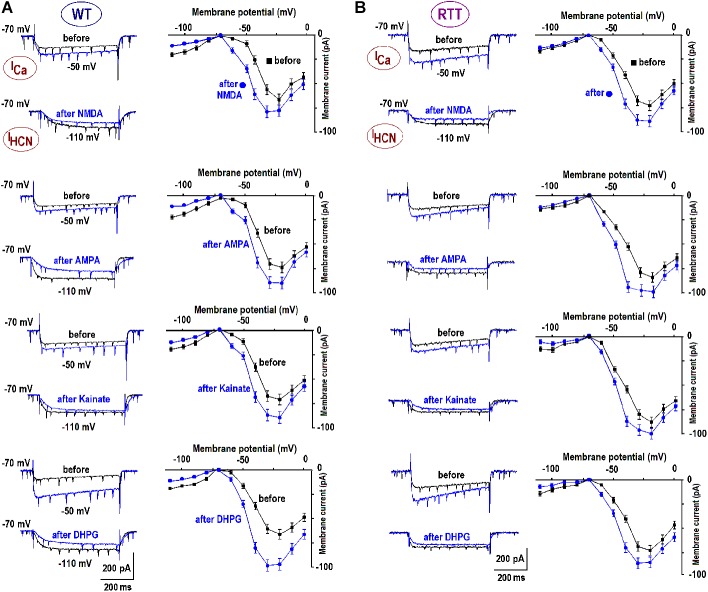
Enhancement of calcium currents and depression of HCN currents after GluR agonists. The data were obtained in CA1 cells from WT **(A)** and RTT **(B)** as indicated. Superimposed sample traces present currents evoked by voltage steps from –70 to –50 mV to evoke subthreshold calcium current, and to –110 to maximally activate HCN current, respectively. The traces were measured before and 10 min after the application of GluR agonists (10 μM NMDA; 10 μM AMPA; 1 μM kainate; and 1 μM DPHG). The graphs show mean I–V curves obtained in these experiments. Calibrations are the same in all panels and scale bars are indicated below the last pair of traces.

We previously reported substantial differences in the voltage-dependent properties of hyperpolarization-activated (HCN) and voltage- VSCC currents in WT and RTT (Balakrishnan and Mironov, 2018b). We sought whether and how the characteristics of these channels are modulated by GluR agonists. In the naïve WT slices, HCN-mediated currents were bigger and VSCC currents were smaller than in RTT (**Figure 3** and **Table 3**). Both channels types in WT were activated at more positive potentials. As noted previously (Balakrishnan and Mironov, 2018b), the differences alone can explain higher excitability in RTT. The data in **Figure 3** and **Table 3** show that after exposure to GluR agonists, VSCC currents in WT became bigger and HCN currents got smaller. A selective long-lasting increase in the high-threshold VSCC channel conductance by GluR agonists was observed in isolated hippocampal neurons (Mironov and Lux, 1992) but the underlying mechanisms were not identified.

**Table 3:** Amplitudes of calcium and HCN-currents in CA1 neurons in slices from WT and RTT animals.

	WT			RTT		
		
	I_Ca_	I_HCN_	Number of tests	I_Ca_	I_HCN_	Number of tests
Before	-73 ± 7	-30 ± 4	4	-96 ± 8	-16 ± 4	5
After 10 μM NMDA	-109 ± 7	-20 ± 3		-118.7 ± 8	-12 ± 2	
Before	-75 ± 5	-39 ± 4	4	-97 ± 5	-24 ± 4	6
After 10 μM AMPA	-106 ± 7	-28 ± 4		-127 ± 8	-16 ± 3	
Before	-74 ± 6	-38 ± 3	3	-87 ± 5	-25 ± 4	4
After 10 μM kainate	-123 ± 6	-28 ± 4		-114.0 ± 7	-14 ± 3	
Before	-76 ± 6	-43 ± 3	5	-89 ± 8	-21 ± 3	6
After 10 μM DHPG	-131 ± 6	-10 ± 1		-117 ± 9	-10 ± 2	


*All tests were made in different fresh (naive) preparations. I_*Ca*_ and I_*HCN*_ amplitudes were measured for voltage steps from -70 to -50 and -110 mV, respectively. Mean data ± SEM were obtained from steady state currents at the end of 300 ms-long pulse. I_*Ca*_ and I_*HCN*_ amplitudes in WT and RTT naive slices are significantly different (*P* < 0.05, Mann–Whitney U test). The changes in I_*Ca*_ and I_*HCN*_ amplitudes after the agonists are significantly different in WT at *p* < 0.01 (Student’s *t*-test, first two data columns) and in RTT at *p* < 0.05 (Student’s *t*-test). After the treatments the amplitudes in WT and RTT were no more significantly different (*P* > 0.1, Mann–Whitney U test).*

Changes in HCN and VSCC conductances developed within 5 min after washout of the agonists and maintained throughout the rest of the recordings that often lasted >1 h. After a single challenge to GluR agonists, the next cell patched in the slice showed bigger VSCC and smaller HCN currents. Thus, a single treatment was sufficient to apparently modify all the cells in the slice. The mean data in **Table 3** was therefore obtained for applications made only in naive slices. After GluR agonists the changes in I_Ca_ and I_HCN_ were significantly bigger in WT (*p* < 0.05, Student’s *t*-test), as if in RTT the channels were already potentiated. After the treatments, the amplitudes in WT and RTT became closer (*P* > 0.1, Mann–Whitney U test). A functional role of changes observed may be two-fold. Firstly, the increase in VSCC conductance and slight leftward shift of the activation curve (**Figure 3B**) should promote calcium-dependent glutamate release. Secondly, HCN channels normally dampen dendritic integration of EPSPs through shunting and electrotonic filtering (Tsay et al., 2007; Vaidya and Johnston, 2013). Therefore, a decrease in HCN conductance, together with the increase in VSCC currents, should make neurons more excitable and resemble the RTT phenotype.

**FIGURE 4:**
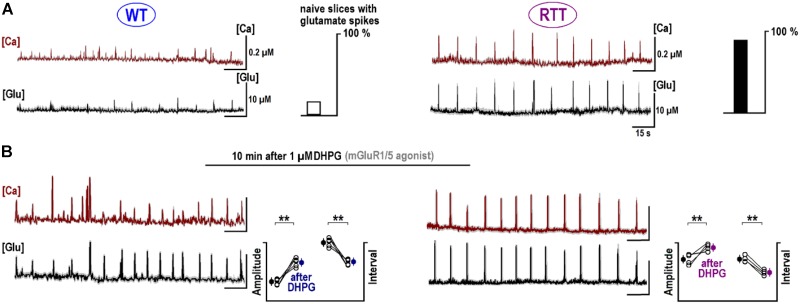
Potentiation of repetitive calcium and glutamate transients after DHPG. The sample traces show the changes in calcium (brown, measured with Fura-2) and ambient glutamate (black, measured with iGluSnFr). Note a close correspondence between both activities. The data are mean responses of 12 neurons in the image field overlaid on gray background showing ± SEM. **(A)** Spontaneous activity in naive slices from the WT and RTT animals. Only 4 out of 25 naive WT slices demonstrated spontaneous glutamate and calcium activities and almost all naïve RTT preparations were “rhythmic” (21 out of 26, the insets give group summary). **(B)** After 1 μM DHPG was applied for 30 s, the changes in intracellular calcium and glutamate were measured using the same ROIs. Group summary in the insets show changes in the amplitude and interval between glutamate spikes in WT (left) and RTT (right). The data before and after drug applications are significantly different at *p* < 0.01 (^∗∗^) and *p* < 0.05 (^∗^), Student’s *t*-test. Calibration bars: 15 s, 10 μM (glutamate imaging); and 0.2 μM (calcium imaging).

Glutamate receptors first increased the neuronal activity and several seconds later the ambient glutamate was raised (**Tables 1, 2**, the third row). Such relatively long-lasting (10–20 s) glutamate elevations could be optimal for activation of metabotropic GluR. We tested mGluR1/5 agonist DHPG and it reliably induced glutamate spikes in WT and potentiated them in RTT (**Figures 1, 2**, the bottommost panels). Imaging glutamate and intracellular calcium with Fura-2 (a read-out of neuronal activity in the slice) showed similar patterns in naïve slices (**Figure 4A**). A correspondence between [Glu]_out_ and [Ca]_in_ changes again underlines close relation between the neuronal bursts and glutamate spikes. After challenge to DHPG, glutamate, and calcium spikes became both markedly bigger and more frequent in the WT slice that initially showed weak periodic activity (left panel in **Figure 4B**). In RTT slices the activities were also significantly potentiated (right panel in **Figure 4B**). The insets in **Figure 4B** present group summaries of these experiments.

**FIGURE 5:**
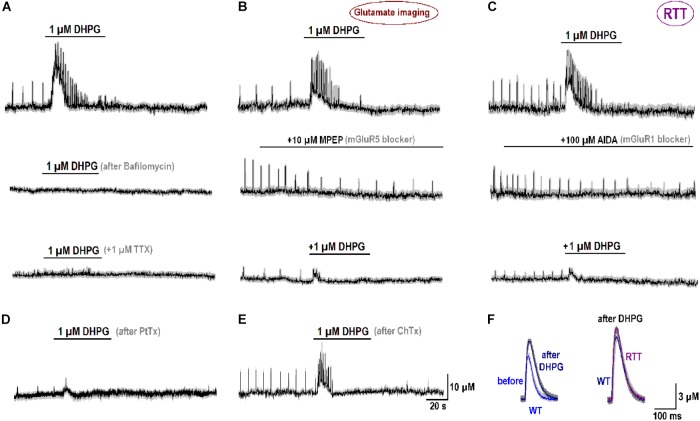
Specificity of mGluR1/5 effects. **(A)** Glutamate release induced 1 μM DHPG (mGluR1/5 agonist; top trace, “control”), The effects was abolished after treatment for 30 min with 1 μM bafilomycin (a blocker of vesicular glutamate uptake, second trace) and 1 μM tetrodotoxin (TTX, bottom trace). **(B)** mGluR5 blocker MPEP diminished amplitude and frequency of spontaneous glutamate spikes (middle panel) and the effects of DHPG (top vs. bottom traces). **(C)** mGluR1 blocker AIDA suppressed glutamate spikes (middle panel) and reduced the effects of subsequent application of DHPG (top vs. bottom traces). **(D)** After treatment of the slices for 24 h with 1 μM/ml PtTx (1 μg/ml), the amplitude and frequency of spontaneous glutamate transients and a stimulatory effects of DHPG were significantly reduced (*n* = 4). **(E)** Neither glutamate spikes nor their augmentation by DHPG were modified after 24 h with Cholera toxin (ChTx, 1 μg/ml). **(F)** Modifications of single glutamate spikes after DHPG. The data were collected using fast imaging (acquisition time, 2 ms). The left panel compares glutamate spikes obtained in WT slices that initially showed weak activity. The traces before and 10 min after DHPG are overlaid. Their comparison clearly indicates increase in the amplitude and decay after the treatment. The right panel compares typical glutamate spikes in WT and RTT that after DHPG became virtually identical.

After treatment of naïve RTT slices with 1 μM bafilomycin (*n* = 3), a blocker of glutamate replenishment in synaptic vesicles, DHPG effects were abolished (middle trace in **Figure 5A**). They were also completely blocked by 1 μM tetrodotoxin (bottom trace in **Figure 5A**). Both observations are in accord with presynaptic source of glutamate. The antagonists to mGluR5 (MPEP) and mGluR1 (AIDA) suppressed glutamate spikes (middle traces in **Figures 5B,C**). They decreased the amplitude of glutamate spikes from 20.1 ± 1.2 to 6.3 ± 1.1 μM (MPEP) and from 21.5 ± 1.5 to 6.1 ± 0.5 μM (AIDA), respectively (*n* = 4, *p* < 0.05, Student’s *t*-test). In the presence of blockers, DHPG-induced elevation of ambient glutamate decreased from 14.2 ± 6.3 (control) to 7.0 ± 1.1 μM (MPEP) and from 16.6 ± 2.4 (control) to 5.1 ± 1.0 μM (AIDA; *n* = 4, *p* < 0.05, Student’s *t*-test for both antagonists). A partial blockade by either drug is consistent with mGluR1 and mGluR5 effects at oriens/alveus interneuron synapses (Le Vasseur et al., 2008).

What subcellular targets can be activated by DHPG remains an open and complex issue that needs further detailed studies. It is yet to mention the results obtained with G-proteins. After incubation of RTT slices with the G_i_ inhibitor Pertussis toxin (PtTx, 1 μg/ml for 24 h), glutamate spikes were markedly suppressed (**Figure 5D**). Their amplitude (4.0 ± 0.5 μM) was much lower than in the control (16.2 ± 4.2 μM) and the interval between them (20.1 ± 2.2 s) was almost twice as big as in the control (12.2 ± 1.1 s; *P* < 0.05, Mann–Whitney U test for both variables). DHPG-induced glutamate release after PtTx treatment was much smaller (1.5 ± 0.3 μM) than in the control (19.5 ± 1.5 μM). On the contrary, stimulation of G_s_ proteins with Cholera toxin (ChTx, 1 μg/ml for 24 h) had no effect (**Figure 5E**). The amplitudes of glutamate spikes were 15.3 ± 3.1 μM vs. 16.2 ± 3.2 μM in the control and DHPG effects had compatible amplitudes (22.3 ± 3.0 μM vs. 24.4 ± 2.5 μM in the control, *n* = 4, *P* > 0.1). Such effects remind of the data obtained in cerebellar granule cells, where G_i,_ but not G_s,_ proteins participated in mGluR1/5 mediated responses (Fagni et al., 2000).

DHPG also modified the time-course of glutamate spikes (**Figure 5F**) resolved with fast imaging (acquisition time, 2 ms). In WT slices with weak activity the mean amplitude of single glutamate spikes increased from 6.2 ± 0.4 to 10.3 ± 0.5 μM (**Figure 5F**, the left couple of overlaid traces) and the decay time-constant slowed down from 65 ± 5 to 105 ± 7 ms (*n* = 3, *p* < 0.05, Student’s *t*-test). The right couple of traces compare the glutamate spikes in WT and RTT after DHPG. Mean amplitude and kinetics were nearly identical (*n* = 4 for both, *P* > 0.1, Mann–Whitney U test). The data in **Figure 5** hence allows suggesting mGluR1/5 as a major sensor of changes in ambient glutamate. The respective elements downstream obviously involve complex signaling pathways and we hope to identify them in future examinations.

**FIGURE 6:**
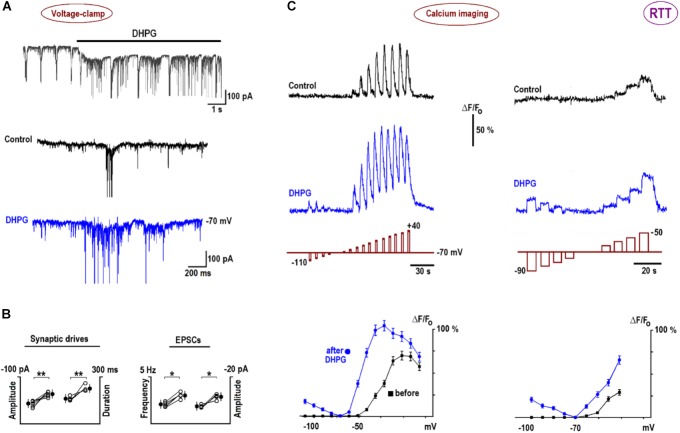
DHPG effects on neuronal activity and calcium influx in RTT. **(A)** DHPG induced an inward current and potentiated excitatory synaptic currents and drives in whole-cell voltage-clamp experiments. **(B)** Group summary of these experiments. **(C)** Potentiation of voltage-triggered calcium increases by DHPG (the voltage steps are depicted below respective traces; note different pulse durations and voltages in the left and right panels). Calcium transients were measured before and 10 min after application of 1 μM DHPG and peak [Ca]-V dependencies are plotted in the bottommost graphs in panel **(C)**. Note calcium increases during hyperpolarizing steps after DHPG, which can be ascribed to activation of calcium-permeable TRPC channels (see also text). Calcium imaging in WT is shown in **Supplementary Figure S2B**.

In voltage-clamp experiments, DHPG induced a slow inward current (**Figure 6A**, mean amplitude–102 ± 7 pA, *n* = 6) and reinforced synaptic and bursting activities. **Figure 6B** presents group summary. The calcium transients reproduced measured I–V curves (**Figure 3**) well. The increases in depolarization-evoked transients after DHPG were in around the same proportion as those observed for VSCC currents (*n* = 6, *p* < 0.01, Student’s *t*-test). Longer pulses in the subthreshold voltage range distinctly showed potentiation of calcium transients after DHPG (the rightmost panels in **Figure 6C**). The increase in VSCC conductance in the subthreshold voltage range is indicative of potentiation in membrane excitability and synaptic transmission (Christie et al., 2010). After DHPG, calcium responses appeared also during hyperpolarization steps (**Figure 6C**) that could be mediated by calcium-permeable TRPC channels activated after DHPG (El-Hassar et al., 2011).

**FIGURE 7:**
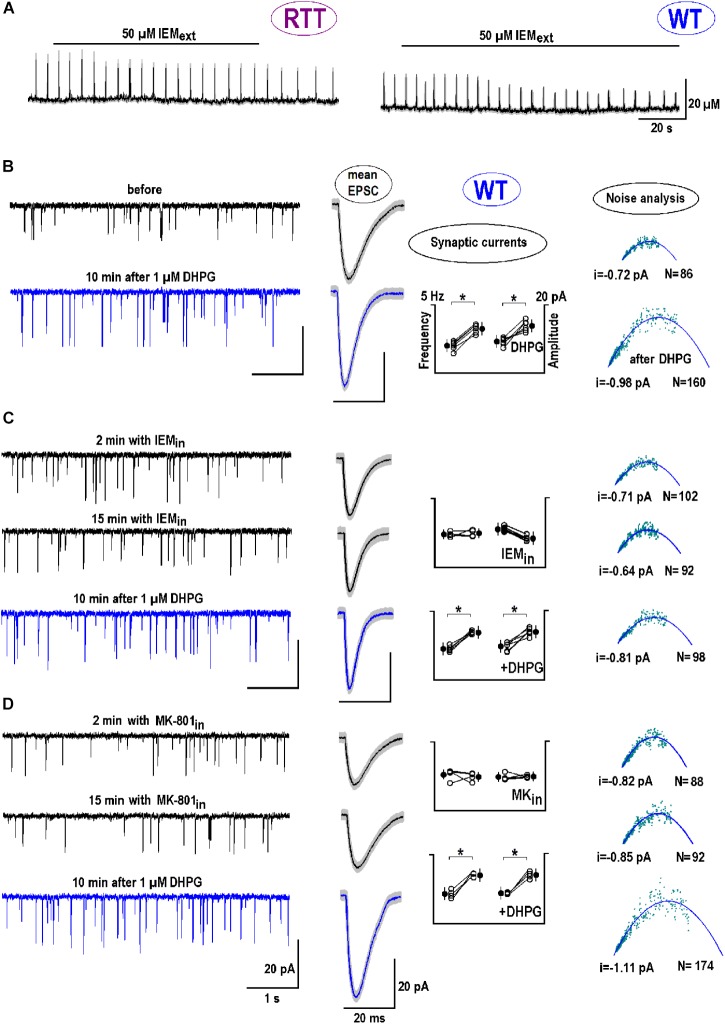
The postsynaptic effects of DHPG: Calcium-permeable (CP)-AMPAR channels. **(A)** Bath application of CP-AMPAR antagonist IEM-1925 (50 μM) depressed glutamate spikes in RTT (spontaneous, the left trace) and WT (DHPG-induced, the right trace). **(B)** The two traces on the left show sample EPSC recordings in WT before and 10 min after DHPG (1 μM for 30 s). The next two smooth traces are mean EPSCs obtained from 30 single events and overlaid upon SEM (a gray background). Next is a group summary for changes in frequency (*f*) and amplitude (*A*) of synaptic currents due to DHPG. The last column presents a non-stationary noise EPSC analysis (see section “Materials and Methods” for details). The outcome was the single channel current (*i*) and the number of channels (*N*) at the postsynapse. From the pool of data sampled in one neuron, we potted the variance (*σ^2^*) against mean current (*I*). Mean-square fitting of *σ^2^*-*I* plots by parabolas returned mean *i* and *N* values at the “‘average” postsynapse. All data were evaluated with Student’s *t*-test with confidence levels of *p* < 0.05 (^∗^). The data in panel **(B)** serves as a “control” of DHPG effects and shows significant changes in all parameters (*f* and *A, i* and *N*; *n* = 6, *p* < 0.05). **(C,D)** The neurons were patched in other and internally perfused with the antagonists to CP-AMPAR (50 μM IEM-1925, **C**) and NMDAR (10 μM MK-801, **D**), respectively. DHPG was then applied for 15 min. **(C)** Cytoplasmic IEM decreased did not change *f* and *A, i* and *N* significantly (*p* < 0.05, *n* = 4). After DHPG both *f* and *A* significantly changed (*p* < 0.05), but *i* and *N* were not markedly modified (*P* > 0.1, Mann–Whitney U test). **(D)** Cytoplasmic MK-801 did not change *f* and *A, i* and *N* (*p* > 0.1, *n* = 4). The effects of DHPG in this case were around the same as in the control (*P* > 0.1, Mann–Whitney U test). **Supplementary Figure S3** shows the postsynaptic changes in WT and RTT after stimulation of ionotropic GluR agonists.

mGluR1/5 mediate chemical long-term potentiation (LTP) at oriens/alveus interneuron synapses of the mouse hippocampus through CP-AMPARs (Le Vasseur et al., 2008). The specific antagonist to CP-AMPAR, IEM-1925 (Tikhonov et al., 2000), decreased the amplitude of glutamate spikes in naive RTT slices from 20.2 ± 3.2 to 12.3 ± 2.0 μM and diminished their frequency from 0.26 ± 0.03 to 0.15 ± 0.02 Hz (**Figure 7A**, left trace; *n* = 5, *p* < 0.05, Student’s *t*-test). For glutamate spikes established in WT slices after DHPG (**Figure 7A**, right trace), IEM-1925 decreased both their amplitude (from 19.3 ± 4.2 to 9.1 ± 3.2 μM) and frequency (from 0.29 ± 0.02 to 0.21 ± 0.02 Hz; *n* = 4, *p* < 0.05, Student’s *t*-test).

We next examined the role of CP-AMPAR antagonist in dendritic integration and analyzed EPSCs. DHPG increased the amplitude and frequency of EPSC from -9.3 ± 3.4 to -14.6 ± 3.2 pA and from 5.8 ± 0.67 to 7.6 ± 0.72 Hz, respectively (*n* = 6, *p* < 0.05, Student’s *t*-test). The mean synaptic currents became bigger and faster after exposure to DHPG (**Figure 7B**, blue vs. black mean traces in the middle column). Observed sharpening of EPSC is in line with the function of HCN channels to dampen dendritic integration through shunting (Tsay et al., 2007; Vaidya and Johnston, 2013). In other neurons the antagonists to CP-AMPAR (**Figure 7C**) and NMDAR (MK-801, **Figure 7D**) were applied intracellularly. Both blockers did not significantly change EPSC frequencies and amplitudes (*n* = 4 for both, *p* > 0.1, Student’s *t*-test) and did not modify the effects of DHPG (*n* = 4 for both, *P* > 0.1, Mann–Whitney U test; the changes appeared in the same proportion as in the control).

Postsynaptic changes were further analyzed using a non-stationary noise analysis (Methods). DHPG reliably increased both the single channel conductance (*i*) and the number of postsynaptic channels (*N*) (**Figure 7B**, rightmost panel, *n* = 6, *p* < 0.01 for both parameters, Student’s *t*-test). Intracellular IEM did not significantly modify *i* and *N* (*n* = 4, *p* > 0.1, Student’s *t*-test), but their changes after DHPG were occluded (*P* < 0.01 vs. control, Mann–Whitney U test). With MK-801 *i* and *N* values significantly increased after DHPG (**Figure 7D**) in the same proportion as in the control (*P* > 0.1 vs. control, Mann–Whitney U test). **Supplementary Figure S3** extends the results of non-stationary noise analysis for other GluR agonists in WT and RTT.

To estimate the effects of synaptic modification after DHPG, assume that a doubling in a number of postsynaptic AMPA channels reflects the insertion of CP-AMPAR. They have 2-fold bigger conductance, which should increase average postsynaptic single channel currents by 50%, close to what is observed after DHPG application (**Figures 7B,D**).

**FIGURE 8:**
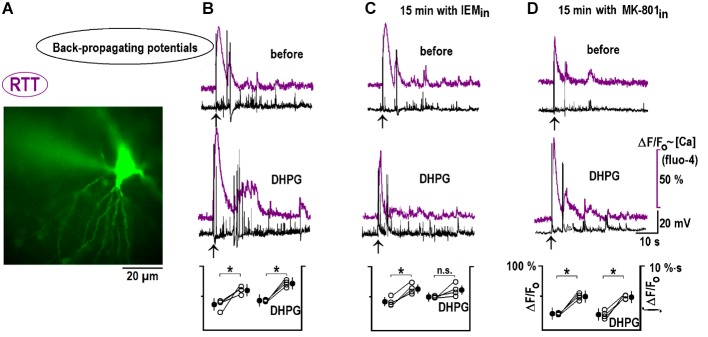
DHPG modifies responses to back-propagating potentials in RTT. **(A)** Spatial distribution of Fluo-4 intensity at the peak of response to back-propagating potentials (bAP). **(B)** Typical responses before and after DHPG (top and middle traces, respectively). Mean calcium responses (violet) and voltage trajectories (black) were measured before and 10 min after DHPG (1 μM for 30 s). Group summary at the bottom gives mean peak Fluo-4 transients during bAPs and fluorescence integral over 2 min after the stimulation (a measure of intensity of spontaneous afterdischarges induced by bAPs). The same protocol was applied to other slices where CA1 neurons were perfused intracellularly for 15 min with CP-AMPAR **(C)** and NMDAR **(D)** blockers as indicated. The data were evaluated with Student’s *t*-test with confidence levels of *p* < 0.05 (^∗^). The differences are significant, except non-significant (n.s.) changes in ∫ (Δ*F/F*_0_) *dt* after DPHG in panel **(C)**. All calibration bars and notations in corresponding panels are the same and listed only once on the right. bAPs in WT are shown in **Supplementary Figure S2B**.

Back-propagating actions potentials can deliver important information about dendritic inputs (Wu et al., 2012). We recorded them in the whole-cell current-clamp mode, together with calcium changes at the apical dendrites. CA1 neurons maintained excitation for ∼1 min after bAPs and calcium transients mirrored the changes in neuronal activity (**Figure 8B**). 1 μM DHPG reinforced these bAP-induced changes (**Figure 8B**). After 15 min with 50 μM IEM inside, bAP-induced responses did not change and the effects of DHPG were occluded (**Figure 8C**). A blockade of postsynaptic NMDAR with intracellular MK-801 did not modify bAPs-induced postactivity and its modulation by DHPG (**Figure 8E**). A group summary for all described experiments is given at the bottom of **Figure 8**.

## Discussion

We previously applied imaging of ambient glutamate in hippocampal slices from Mecp2-deficient mice (Balakrishnan and Mironov, 2018a,b) to test the hypothesis whether improper glutamate handling may be responsible for aberrant neuronal phenotype in RTT. Unexpectedly, we routinely observed repetitive glutamate transients (spikes) in the CA1 area in hippocampal slices prepared from RTT animals. This can represent another important hallmark, because in naïve WT slices such spikes were virtually absent. Repetitive glutamate transients utilize the two deviations of CA1 neurons in RTT – higher intrinsic excitability as determined by altered voltage-dependencies of HCN and VSCC channels and slower glutamate uptake. The observations are supported by the modeling (Balakrishnan and Mironov, 2018a) showing that a combination of more intense presynaptic glutamate release and slower glutamate clearance creates sufficient conditions for GIGR. It relies upon recurrent excitation that leads to further presynaptic release and establishes brief synchronous transients within the whole CA1 network.

Enhanced excitability in CA1 RTT neurons due to smaller HCN currents and bigger VSCC currents can be countered by appropriate changes in the surface potential that moves the activation curves along the voltage axis (Balakrishnan and Mironov, 2018b). For example, their leftward shift in the alkaline solutions (pH 8.4) enhances excitability in WT slices and transforms the activity into an RTT pattern. Acidic solutions (pH 6.4) and elevated Mg^2+^ shift the activation curves rightward and dampen excitability producing apparent “WT phenotype” in RTT slices. Mg^2+^ has indeed been empirically found to relieve RTT patients from the most severe symptoms (Egger et al., 1992). A shortcoming of the treatment is that the effects of surface potential cannot be maintained and restoration of initial conditions quickly reverses the effects of pH and Mg^2+^.

We sought for other factors that may induce long-term modifications in the activity in RTT and turned to glutamatergic receptors. GluR agonists activated a massive increase in ambient glutamate (**Figures 1, 2**). The activity was then depressed for ∼1 min and restored after wash-out of the agonists, but at higher activity levels. In WT, regular glutamate spikes appeared whose pattern resembled a typical behavior of naïve RTT slices. After GluR agonists in RTT the amplitude and frequency of glutamate transients increased. In both preparations the effects were long-lasting (>1 h). Brief augmentation with subsequent depression of activity, and the establishment of rhythmic pattern after recovery from brief stimuli were previously noticed in the hippocampal (Toloe et al., 2014) and brainstem networks (Mironov, 2008, 2009, 2013).

To our knowledge, actual glutamate levels in nervous tissue have been never measured at sufficient temporal and spatial precision, except in few recent studies (Marvin et al., 2013; Parsons et al., 2016). GluR agonists increased ambient glutamate to ∼10 μM in WT for ∼10 s and for ∼20 μM for ∼20 s in RTT (**Tables 1, 2**). We believe that such effects employs GIGR mechanism and GluR agonists (AMPA, NMDA, kainate, and DHPG) act as the triggers. They excite neurons (in RTT to more extent) that activate glutamate release. The GIGR mechanism requires an intact glutamatergic network and is abolished after elimination of presynaptic glutamate release (Balakrishnan and Mironov, 2018a; see also the inhibition of DHPG effects by bafilomycin and tetrodotoxin in **Figure 5A**). GluR agonists are thus only the tools that activate GIGR and subsequent events develop autonomously. This can explain stereotypic responses observed for all GluR (**Figures 1, 2**).

We propose that the long-lasting effects in postsynaptic neurons are mediated by mGluR1/5. The receptors are located perisynaptically (Baude et al., 1993), coupled to G_q/11_ and phosphoinositide cascades that regulate postsynaptic excitability. mGluR1/5 is implicated in a number of physiological and pathological responses such as modulation of neuronal excitability, synaptic transmission, LTP, epileptiform activity, and postischemic injury. The most immediate effects of mGluR agonist DHPG on CA1 pyramidal neurons are membrane depolarization and increased AP firing. Immediate effects implicate the inward current mediated by a non-specific cationic conductance (Crépel et al., 1994; Mironov, 2008). In this study we also observed inward current after DHPG (**Figure 6A**). The voltage-clamp and calcium imaging data (**Figure 6B**) is consistent with the activation of canonical calcium-permeable TRPC1 channels (El-Hassar et al., 2011).

Postsynaptic mGluR provoke paroxysmal bursts in CA1 through inhibition of glutamate transport (Molinari et al., 2012) that should increase ambient glutamate. The bursts require a synaptic release of glutamate and extrasynaptic GluR receptors activated by transmitter spillover. The authors propose that the postsynaptic mGluR are tonically activated by the rise in ambient glutamate concentration and strongly contribute to burst generation that implies a positive feedback between the bursts and glutamate release. This scenario may work in RTT where naïve slices show elevated glutamate in CA1 due to diminished glutamate uptake (Balakrishnan and Mironov, 2018a).

The data is congruent with localization of the mGluRs in the neurons. At present, we cannot exclude their functional presence in the astrocytes, however. In a common model of tripartite synapses, the astrocytes can feel glutamate release during synaptic transmission and report this using mGluR1/5 (cf. Panatier and Robitaille, 2016 for recent review). Astrocytes represent a major sink of ambient glutamate. After DHPG the glutamate spikes became bigger and slower (**Figure 5F**) that may reflect mGluR1/5 effects in astrocytes. Single astrocytes contact around thousands of synapses and hundreds of dendrites from several neurons, which presumes intense communication between neurons and glia. This hypothesis was and still remains a hot topic in neuroscience. Perhaps it can be properly tackled in future, when the sensors of mGluR activity and glutamate in fine astrocytic processes (now considered as real players in integration of neuronal and glial activities), will become available.

Stimulation of metabotropic receptors modifies postsynaptic AMPARs. The result reminisces with their role in the brainstem respiratory neurons (Mironov, 2013). Brief excitation of the respiratory network *in vitro* induces or reinforces repetitive activity, depending on its initial state – silent or rhythmic. mGluR1/5 enhances AMPA-dependent glutamatergic transmission that strengthens the coupling between the respiratory neurons and augments the output. When a network enters the oscillatory state, the activity is further maintained through periodic discharges. A close correspondence between long-term modifications of hippocampal and brainstem networks tempts to speculate that transition from “silent” to oscillatory network may mechanistically represent a common response after brief periods of excitation.

Modifications in HCN and VSCC currents in WT CA1 neurons after DHPG make them resemble putative currents in naive RTT CA1 neurons (**Figure 3**). HCN channels normally act as an inhibitory constraint of synaptic integration and long-term plasticity in CA1 dendrites. Their down-regulation after exposure to DHPG in WT and RTT (to less extent in this case), should diminish the effects of active shunt conductance and increase temporal integration of distal EPSPs leading to an additional increase in excitability.

Back-propagating potentials are important for synaptic plasticity (Magee and Johnston, 1997; Stuart and Häusser, 2001). They are able to trigger Ca^2+^ entry by activating voltage dependent Ca^2+^ channels (Sabatini and Svoboda, 2000), activate NMDA receptors and facilitate the removal of intrinsic Mg^2+^ blockade (Nevian and Sakmann, 2004; Wu et al., 2012). After bAPs, the neuronal activity and accompanying calcium signals were enhanced for 1–2 min in naïve slices and further potentiated after DHPG treatment (**Figure 8**). Such changes can also be ascribed to observed modification in HCN and VSCC conductances. mGluR1/5 mediates chemical LTP at oriens/alveus interneuron synapses of the mouse hippocampus that also utilizes CP-AMPAR (Le Vasseur et al., 2008). In RTT, naïve excitatory synapses onto hippocampal pyramidal neurons were up-regulated through elevated expression of CP-AMPAR (**Figure 7**). This effect should additionally promote hyperexcitability in RTT.

Recent evidence highlighted the role of mGluR in RTT. For example, mGluR5 underlie abnormal protein-synthesis-dependent synaptic plasticity in the hippocampus (Gogliotti et al., 2016; Tao et al., 2016). mGluR5 is predominantly expressed in CA1 pyramidal cells (Baude et al., 1993), but they also display mGluR1 mRNA (Shigemoto et al., 1992). In the CA1 neurons of Mecp2 knockout, a subset of ribosome-bound mGluR1 mRNAs, whose sequence significantly overlapped with FMRP direct targets and autism genes (Tao et al., 2016). Such anomalies were proposed to contribute to deficits in LTP and cognitive impairments in neurodevelopmental disorders associated with intellectual disability and autism.

## Conclusion

In summary, the imaging of ambient glutamate showed that brief challenge to GluR agonists induces a global release of ambient glutamate in the CA1 hippocampus. After this, the activity in WT slices apparently acquired RTT phenotype. The effects correlate with the modulation of HCN and calcium conductances and the incorporation of new AMPA receptors. All these factors converge and increase postsynaptic excitability. Our data posits mGluR1/5 as the putative molecular candidate to sense brief glutamate elevations. The target can be used to elaborate pharmacological paradigms to counter or ameliorate consequences of Mecp2-deletion.

## Author Contributions

SM designed the experiments and figures, evaluated the tests, wrote the manuscript, and acquired funding. SB performed patch-clamping and imaging, and validated the results.

## Conflict of Interest Statement

The authors declare that the research was conducted in the absence of any commercial or financial relationships that could be construed as a potential conflict of interest.
