# Supplementary material for: CA1 Neurons Acquire Rett Syndrome Phenotype After Brief Activation of Glutamatergic Receptors: Specific Role of mGluR1/5
Source: Front Cell Neurosci. 2018 Oct 17;12:363. doi: 10.3389/fncel.2018.00363 (PMC6199391; doi:10.3389/fncel.2018.00363)
Supplement: Supplementary file 1 [file Data_Sheet_1.docx]

**Supplementary information**


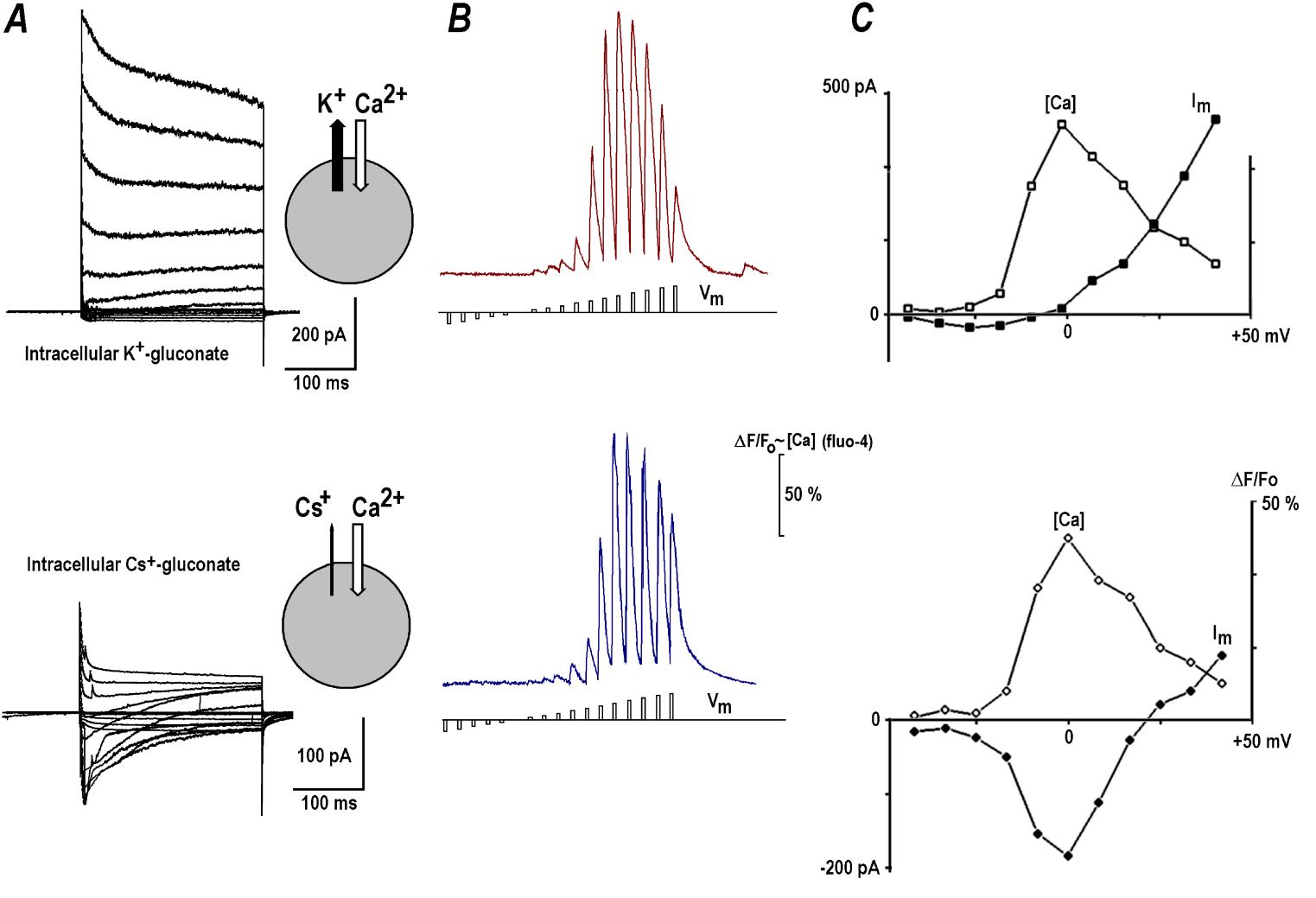


**Fig. S1**. Fluorescence and voltage-clamp measurements of calcium influx

***A*** – Membrane currents were measured in CA1 neurons from WT animals during voltage steps from the holding potential -70 mV with +10 mV increment. With high K^+^-intracellular solution the current was largely outward (*top*). With Cs^+^ + TEA (tetraethylammonium) in the pipette, the current was inward at intermediate depolarization (*bottom*), and became outward only during voltage steps more positive than +20 mV. ***B*** – Transient changes in intracellular calcium measured with fluo-4. The data were obtained during a voltage-clamp experiments shown in (***A***) with voltage protocols schematically shown under the traces. The calcium increases were evoked only by depolarizing steps, indicating a negligible permeability of HCN channels to calcium. The voltage dependence of calcium transients was similar for the two different intracellular solutions. ***C*** – Peak calcium levels and membrane currents. Note the good correlation between [Ca]-V and I-V plots measured with intracellular Cs^+^ + TEA solution that deviate only at positive voltage pulses. Although smaller calcium current in this case was masked by the outward current; calcium entry was yet visible as the increase in fluo-4 fluorescence.

**
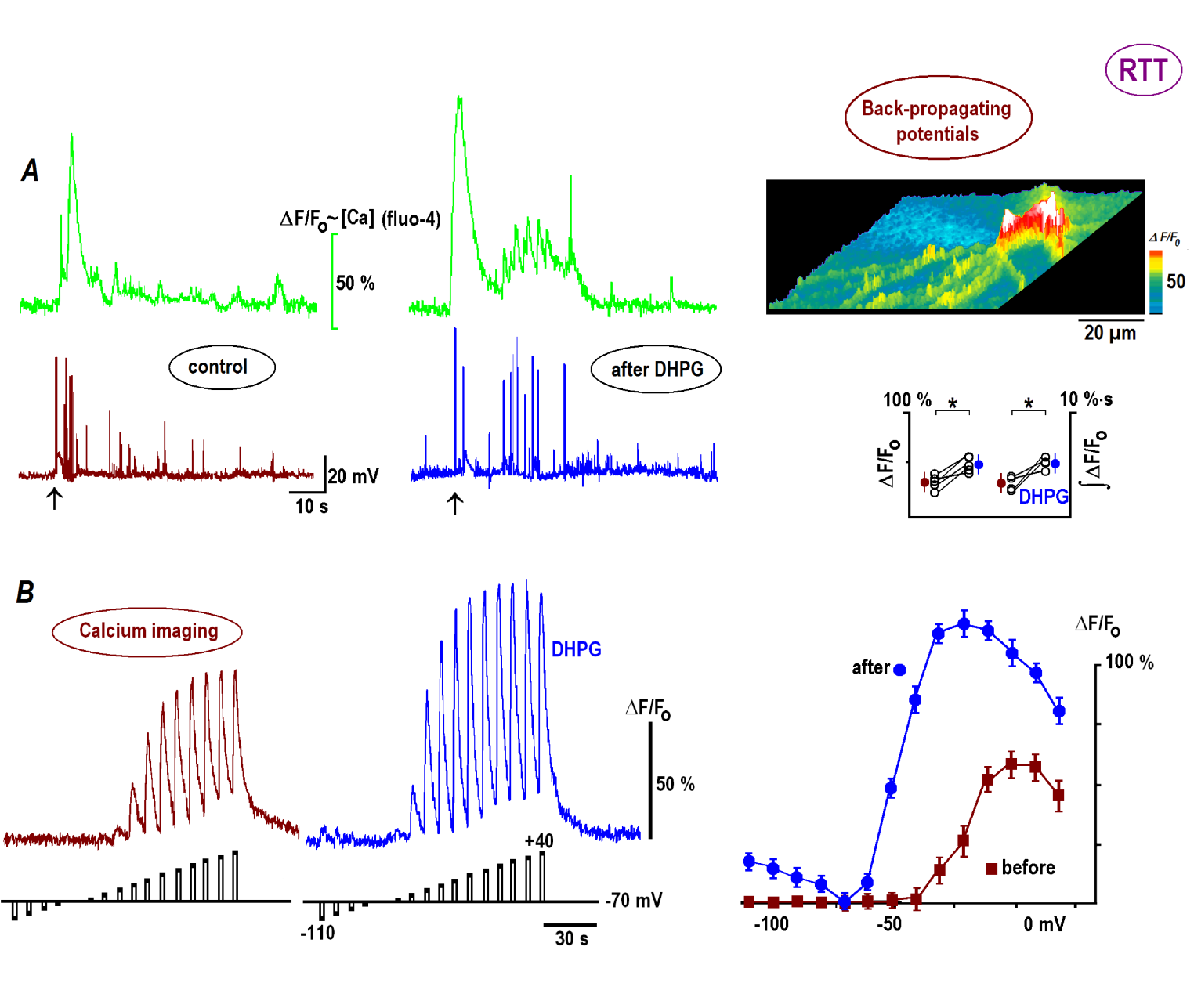
**

**Fig. S2.** The effects of mGluR1/5 stimulation in CA1 cells from RTT animals

***A*** – Back-propagating potentials (bAPs) were evoked by 8 pulses delivered at 100 Hz. Calcium changes were measured with fluo-4. The spatial calcium distribution at the peak of response is depicted in the inset on the right and a group summary is shown below. The data were evaluated before and after applications of DHPG with Student’s *t* test with confidence levels of *p* < 0.05 (*). For bAPs-evoked events, peak changes and integral of fluorescence over 2 min after bAPs (a measure of intensity of spontaneous afterdischarges) were compared. ***B*** – Voltage-dependent calcium influx and its augmentation by DHPG. *Left* – experimental traces for the voltage protocol depicted below, *right* - [Ca]-V dependencies. Note calcium increases during hyperpolarization steps after DHPG that might have invoked non-selective TRPC channels, permeable to calcium.


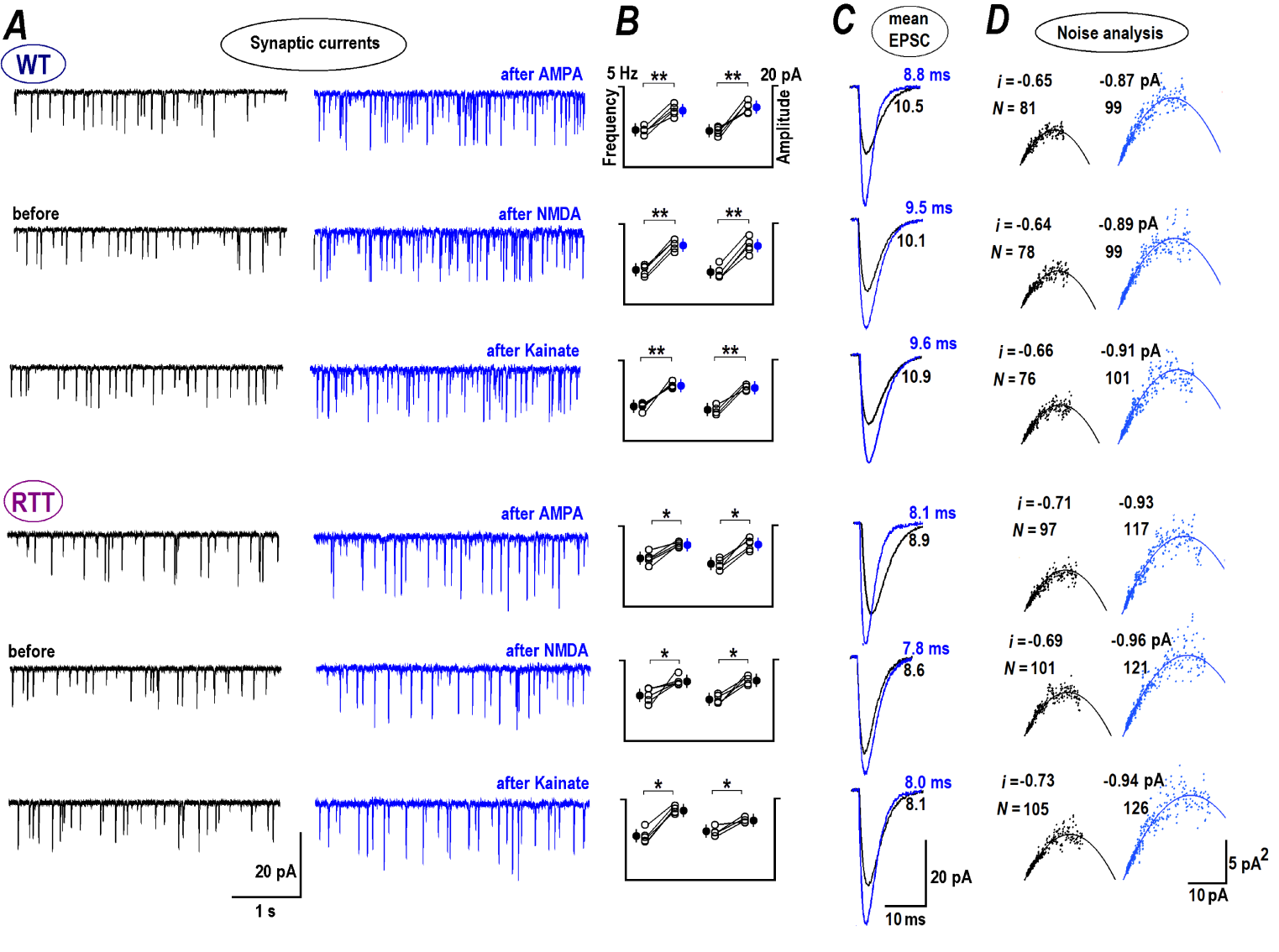


**Fig. S3.** Changes in postsynaptic currents after agonists to ionotropic glutamate receptors.

The data were obtained in CA1 cells from WT and RTT animals as indicated. ***A*** – The two traces in each row illustrate EPSCs before and 10 min after application of 10 µM AMPA, 10 µM NMDA and 1 µM kainate. ***B*** – Group summary for changes in EPSC frequency and amplitude after GluR agonists. ***C*** – Mean EPSCs were obtained from 30 single events before and after application of GluR agonists. The time constants listed near corresponding traces were estimated by fitting decays with single exponential function. ***D*** - Non-stationary noise analysis of EPSCs. Shown are the plots of variance (*σ^2^*) vs. mean current (*I*) for the pool of experimental data sampled on the left. The plots were mean-square fitted by parabolas whose parameters delivered mean single channel current (*i*) and the number of channels (*N*) involved in the generation of EPSCs. All mean values had <± 10% relative SEM.
